# Molecular mechanisms of plant growth promotion for methylotrophic *Bacillus aryabhattai* LAD

**DOI:** 10.3389/fmicb.2022.917382

**Published:** 2022-10-24

**Authors:** Chao Deng, Xiaolong Liang, Ning Zhang, Bingxue Li, Xiaoyu Wang, Nan Zeng

**Affiliations:** ^1^College of Land and Environment, Shenyang Agricultural University, Shenyang, China; ^2^Key Laboratory of Pollution Ecology and Environmental Engineering, Institute of Applied Ecology, Chinese Academy of Sciences, Shenyang, China; ^3^College of Bioscience and Biotechnology, Shenyang Agricultural University, Shenyang, China

**Keywords:** carbohydrate-active enzymes, sequencing, rhizosphere, growth promoting, indole acetic acid

## Abstract

Plant growth-promoting rhizobacteria (PGPR) can produce hormone-like substances, promote plant nutrient uptake, enhance plant resistance, inhibit the growth of pathogenic bacteria, and induce plant resistance to biotic and abiotic stresses. *Bacillus* is one of the most studied genera that promote plant root development. Since its discovery in 2009, *B. aryabhattai* has shown promising properties such as promoting plant growth and improving crop yield. However, the mechanisms of *B. aryabhattai* promoting plant growth remain to be investigated. In this study, the chromosome of *B. aryabhattai* strain LAD and five plasmids within the cell were sequenced and annotated. The genome, with a length of 5,194,589 bp and 38.12% GC content, contains 5,288 putative protein-coding genes, 39 rRNA, and 112 tRNA. The length of the five plasmids ranged from 116,519 to 212,484 bp, and a total of 810 putative protein-coding genes, 4 rRNA, and 32 tRNA were predicted in the plasmids. Functional annotation of the predicted genes revealed numerous genes associated with indole-3 acetic acid (IAA) and exopolysaccharides (EPSs) biosynthesis, membrane transport, nitrogen cycle metabolism, signal transduction, cell mobility, stress response, and antibiotic resistance on the genome which benefits the plants. Genes of carbohydrate-active enzymes were detected in both the genome and plasmids suggesting that LAD has the capacity of synthesizing saccharides and utilizing organic materials like root exudates. LAD can utilize different carbon sources of varied carbon chain length, i.e., methanol, acetate, glycerol, glucose, sucrose, and starch for growth and temperature adaptation suggesting a high versatility of LAD for thriving in fluctuating environments. LAD produced the most EPSs with sucrose as sole carbon source, and high concentration of IAA was produced when the maize plant was cultivated with LAD, which may enhance plant growth. LAD significantly stimulated the development of the maize root. The genome-based information and experimental evidence demonstrated that LAD with diverse metabolic capabilities and positive interactions with plants has tremendous potential for adaptation to the dynamic soil environments and promoting plant growth.

## Introduction

Plant growth and development is highly dependent on the interactions with other living organisms that habitat the soil ecosystem. These interactions are very complex and critical for maintaining the biodiversity in the below-ground system (Lau and Lennon, 2011; Bever et al., 2013; Shao et al., 2018). Microbes are the most abundant and diverse entities in soil and can directly participate in ecological processes and nutrient cycling. The role of soil microbial community in soil ecosystem functioning and plant production has been widely investigated *via* isolation of culturable microbes and culture-independent techniques (Liu et al., 2019; Roy et al., 2020). However, the soil environment is extremely heterogeneous imposing great challenges on studies of soil microbial ecology.

Roots harbor a rich abundance of biomass and are the crucial organ of the plant to absorb water and nutrients for the plant. Rhizosphere the narrow region immediately adjacent to the root is the plant root-soil interface and is the hot spot for microbial interactions and cross-kingdom interactions between plants and microbes (Chaparro et al., 2014; Korenblum et al., 2020). Rhizosphere microbes and the plant may form symbiotic relationships in which the root microbiome utilizes root exudates and secretes compounds benefiting plant growth (Berendsen et al., 2012). Rhizosphere microbes may synthesize antibiotics as required by the plant for suppression of soil-borne pathogens and ultimately enhance the plant health (Mendes et al., 2013; Lazcano et al., 2021). Other rhizosphere microbiome-plant mutualistic interactions include microbiome fixing and providing essential elements (e.g., nitrogen) for plant growth (Moreau et al., 2019). Rhizosphere microbiome may also induce systemic root exudation of metabolites and mediate root-root signaling promoting soil conditioning (Korenblum et al., 2020). Meanwhile, many environmental and biotic factors can influence rhizosphere microbiome-plant interactions making it more complicated to study the mechanism of different rhizosphere microbes promoting plant growth.

Rhizospheric microorganisms could produce hormone-like substances, then promote plant growth and nutrient uptake, inhibit the growth of pathogenic bacteria and induce plant resistance to biotic and abiotic stresses (Ahmed and Hasnain, 2014). For example, inoculation with typical beneficial microbial mycorrhizal fungi not only promotes the secretion of organic acids and phosphatases from the roots of symbiotic plants, but also enhances the ability of plant to activate insoluble phosphates in the soil and promotes the uptake of water and minerals, especially phosphate (Berta, 2000; Erik et al., 2000; Philippe, 2001; Maria, 2005). *Bacillus amylolyticus* strain B3 harbors functional genes capable of directly promoting crop growth, like *yhcX* and *ysnE*, key genes for plant growth hormone (IAA) synthesis; and *AlsS*, *AlsD*, and *AlsR*, synthase genes related to the volatile disease-promoting substance 2,3-butanediol. The phytase synthesis gene *phy* is present intact in the B3 genome, and phytase can catalyze the degradation of phytic acid into inositol, facilitating plant uptake and utilization of nutrients from soil (Idriss et al., 2002; Zhang et al., 2010). The exocrine secretion of *Bacillus subtilis* BS-2 promotes rice growth, increases chlorophyll content in the crop, slows membrane lipid peroxidation in rice, and promotes indoleacetic acid production in the plant (Ma et al., 2018). Bacterial colonization can trigger plant immune reaction, and the interaction between beneficial bacteria and plant immune system is essential for efficient bacterial colonization, survival, and plant growth promoting hormone production (Tzipilevich et al., 2021).

*Bacillus* is the one of the most studied genera among the plant growth promoting rhizobacteria and shows tremendous potential in promoting plant growth (Chen et al., 2007; Tahir et al., 2017; Backer et al., 2018). *Bacillus aryabhattai* are widely distributed in nature but were only discovered in 2009 (Shivaji et al., 2009). More strains of *B. aryabhattai* were isolated from various environments including plant roots, and evaluations of this rhizosphere bacteria have revealed promising properties of this *Bacillus* species for promoting plant growth and improving crop yields thus having attracted plenty of attention from researchers (Bhattacharyya et al., 2017; Park et al., 2017; Ghosh et al., 2018). Mehmood et al. (2021) found that *B. aryabhattai* promoted wheat growth and reduced the effects of salt stress on wheat. However, the mechanism of this rhizobacteria species promoting plant growth remains to be investigated. With the development of sequencing technology, whole genome sequencing of the isolated microbial strains has been widely used to reveal the potential functions of the microbes in enhancing the plant performance (Chu et al., 2020; Chen et al., 2022).

In a recent study, an excellent plant inter-rhizosphere strain, *B. aryabhattai* LAD, was isolated from maize rhizosphere, and the impact of LAD on rhizosphere microbial structure was also explored in the previous study (Deng et al., 2022). LAD showed promising plant growth promoting properties, including nitrogen fixation and phosphorus solubilization and IAA production. Here, we aimed to reveal the genes related to plant growth-promoting functions of this strain through whole genome sequencing and investigate the genetic differences between this strain and similar plant growth promoting rhizobacterial strains *via* comparative genomics. The metabolic and phenotypic traits and habitat-specific adaptations of this strain to maize root promotion were also investigated.

## Materials and methods

### Total DNA extraction and whole genome sequencing

The strain LAD was isolated from maize rhizosphere in our lab, and initial characterization of the newly isolated bacterial strain was performed and the effects of LAD on corn seedlings were also evaluated. In this study, LAD was grown in its optimum growth medium containing 20 g L^−1^ sucrose, 2 g L^−1^ beef extract, 0.4 g L^−1^ KH_2_PO_4_, 0.4 g L^−1^ MgSO_4_·7H_2_O, 0.4 g L^−1^ NaCl, 0.4 g L^−1^ CaSO_4_·2H_2_O, and 2 g L^−1^ CaCO_3_ at 180 rpm and 37°C. The bacterial cells were harvested at the late exponential phase (around 22 h) for total DNA extraction with the extraction kit. The extracted DNA was quantified with Nanodrop 2000 spectrophotometer (Thermo Fisher Scientific, Waltham, MA, United States) and sent out to Shanghai Personal Biotechnology Co., Ltd. for next-generation sequencing of the total DNA. Whole genome sequencing of LAD was performed using on PacBio Sequel (Pacific Biosciences of California, Inc., Menlo Park, CA, United States) and Illumina NovaSeq (Illumina, Inc., San Diego, CA, United States) sequencing platforms, respectively.

### Whole genome annotation

The obtained sequence reads from PacBio platform were assembled into contigs using HGAP4 WGS-Assembler 8.2 and CANU (Chin et al., 2016; Koren et al., 2017). The Illunima reads were employed for correction of the assembled contigs with Pilon 1.22 to get the final complete genome (Walker et al., 2014). GeneMarkS was used for finding protein-coding genes *via* GeneMark.hmm program (Besemer et al., 2001). Transfer RNAs (tRNAs) were predicted using tRNAscan-SE, and the prediction of other non-coding RNAs was performed in Rfam (Lowe and Eddy, 1997; Kalvari et al., 2018). Clustered regularly interspaced short palindromic repeats (CRISPRs) were predicted using CRISPRFinder program (Grissa et al., 2007). PHASTER (Phage Search Tool Enhanced Release) was used for detection of prophages on the genome (Arndt et al., 2016).

### Genomic comparisons

Reference genome sequences of other *Bacillus* strains were retrieved from NCBI GenBank database for comparative genomic analysis of *B. aryabhattai* LAD (Table 1). *B. aryabhattai* is a homotypic synonym of *Priestia aryabhattai*. Pairwise genome comparisons were performed on JSpeciesWS which evaluates whole genome homologies *via* BLAST alignments for determination of the average nucleotide identity (ANI) between genome sequences (Richter et al., 2016). The bacterial pan genome analysis (BPGA) pipeline was used to identify the orthologous pan genome profile among the genomes of the *B. aryabhattai* strains (Chaudhari et al., 2016). Core genome (the set of shared genes by all strains), accessory genome (genes shared by different strains but not by all strains), and unique genes (exclusively belonging to one strain), and pan genome (non-homologous genes) were identified with BPGA. Carbohydrate-active enzymes (CAZy) were characterized based on the CAZy database (Lombard et al., 2014). The whole genomic sequence data of *B. aryabhattai* LAD was deposited to NCBI GenBank, with the accession number of GCA_017743055.1.

**Table 1:** Genome information of 19 strains used in this study.

Organism	GenBank accession	Level	Size (Mb)	Gene
*B. aryabhattai* B8W22	GCF_000956595.1	Contig	1.72	5,135
*B. cereus* group sp. N11	GCF_016483605.1	Contig	1.99	4,923
*B. cereus* group sp. N6	GCF_016483705.1	Contig	1.95	5,104
*B. megaterium* DSM319	GCF_000025805.1	Complete	1.7	4,987
*B. megaterium* NCT-2	GCF_000334875.3	Complete	1.91	4,640
*B. megaterium* ATCC 14581	GCF_006094495.1	Complete	1.9	4,908
*Bacillus* sp. AM1(2019)	GCF_009906915.1	Complete	1.5	5,179
*B. aryabhattai* K13	GCF_002688605.1	Complete	1.8	4,844
*B. aryabhattai* LAD	GCA_017743055.1	Complete	1.64	5,198
*B. aryabhattai* AB211	GCF_001858395.1	Scaffold	1.85	4,682
*B. aryabhattai* AFS075785	GCF_002569785.1	Scaffold	1.78	4,386
*B. aryabhattai* FJ-6	GCF_013372535.1	Contig	1.78	4,153
*B. aryabhattai* B14	GCF_002167185.1	Contig	1.6	5,006
*B. megaterium* BIM B-1314D	GCF_013389435.1	Complete	2	4,822
*B. aryabhattai* S00060	GCF_014138775.1	Scaffold	2.02	5,346
*B. aryabhattai* G25-109	GCF_015845475.1	Contig	1.8	5,378
*B. megaterium* CDC 2008724142	GCF_017086565.1	Complete	1.99	5,274
*B. aryabhattai* ME39	GCF_903971025.1	Contig	1.9	5,611
*B. megaterium* H2	GCF_017352315.1	Complete	2.12	5,322

### *Bacillus aryabhattai* LAD growth under different temperatures and carbon sources

The growth of LAD on different carbon sources and at different temperatures was evaluated with Epoch 2 microplate spectrophotometer (BioTek Instruments Inc., Winooski, VT, United States). LAD cells were inoculated in LB medium and grown at 37°C overnight. The LAD culture was used as inoculum (1%) for growth in the optimum growth medium, but the carbon source in the medium was the same concentration (2%, m v^−1^) of sodium acetate, glycerol, glucose, sucrose, or starch. The growth of LAD on methanol was also examined in the optimum medium but with the carbon source replaced with 0.5% (v v^−1^) methanol. The growth curves of LAD populations on different carbon sources and at different temperatures (20°C and 37°C) were obtained by culturing the cells in 24-well plates and analyzed at a 1-h interval in the Epoch 2 microplate spectrophotometer. Each treatment included three replicates, and the mean cell density was used to plot the growth curves.

### Indole-3-acetic acid production by *Bacillus aryabhattai* LAD

The maize seeds (Dongdan 1,331) were immersed in 10^5^ CFU ml^−1^ LAD cell suspensions and cultivated in the artificial climate room for 8 days. As the control treatment, the maize seeds were immersed in sterilized water and cultured for 8 days under the same conditions. Each treatment included three replicates, and *t*-test was used to reveal the statistical differences between LAD treatment and control. The plant was removed after cultivation, and the liquid culture was concentrated by rotary evaporation and was archived for later use. Liquid chromatography system of Waters ACQUITY UPLC (Milford, MA, USA) with a liquid chromatography column (182.1 mm × 50 mm i.d., 1.7 μm) was used to determine the amount of the produced IAA (Fu et al., 2012). IAA was isolated in a mobile phase consisting of methanol solution (mobile phase A) and 0.1% formic acid aqueous solution (mobile phase B). The gradient elusion was run at a flow rate of 0.2 ml min^−1^ with initial 20% mobile phase A which was increased to 80% in the next 12 min. The volume fraction of mobile phase A was reduced from 80 to 20% in the time range of 12 to 16 min. The injection volume for all samples was 3 μl and the column temperature was 40°C. The mass spectrometer that affiliated with the UPLC system used an electrospray ion source, with a capillary voltage of 0.8 KV in positive ion mode (ESI+), a taper voltage of 25 V, a dissolvent temperature of 650°C, a dissolvent gas flow rate of 1,000 l h ^−1^, and a conical hole back blow 5 l h ^−1^. The collision voltage was set 30 V at 176 > 103 and 15 V at 176 > 130.

### Production of exopolysaccharides

LAD cells were grown in LB medium at 37°C for 24 h before the cells were transferred into the optimum growth medium with different carbon sources, i.e., methanol (0.5, v v^−1^), sodium acetate (2%, m v^−1^), glycerol (2%, m v^−1^), glucose (2%, m v^−1^), sucrose (2%, m v^−1^), and starch (2%, m v^−1^), respectively. The bacterial cells were cultivated at 37°C with the EPSs production being evaluated at 24 and 72 h of the cultivation. For EPSs extraction, 10 ml bacterial culture was centrifuged at 5,000 rpm for 20 min, and the supernatant was transferred to a clean 50 ml centrifuge tube with 2-fold volume of 95% ethanol. The mixture was stirred using a glass rod until clear flocculent precipitation appeared. The mixture was stored at 4°C for 24 h before centrifugation at 10,000 rpm for 15 min. The extracted products, crude EPSs, were air dried at room temperature and weighed for determination of the EPSs production on different carbon sources.

### Impacts of LAD on maize root development

LAD was cultivated in selective nutrient broth at 37°C for 72 h (until OD_600nm_ reached 1.4). The LAD cells were harvested by centrifugation and resuspended in sterile water, and the cell suspension was diluted to a final concentration of 10^5^ CFU ml^−1^ using sterile water. The maize seeds were immersed in the diluted bacterial suspension for 24 h, after which the maize seeds were cultivated in laboratory hydroponics and in the field, respectively. In the control treatment, the maize seeds were soaked in the sterile water for 24 h before being cultivated in laboratory hydroponics and field. The root development of the maize seedlings in hydroponics was measured by a root system analyzer after 14 days, while measurement of the root development of each maize plant grown in field was performed after 60 days (Bucksch et al., 2014). The hydroponic and field cultivation for each treatment included 10 maize plants, respectively. Each treatment was repeated three times. Student’s *t*-test was used to compare the root development under LAD treatment with that of the control to show the statistical differences.

## Results

### Functional annotation of *Bacillus aryabhattai* LAD whole genome

**Figure 1:**
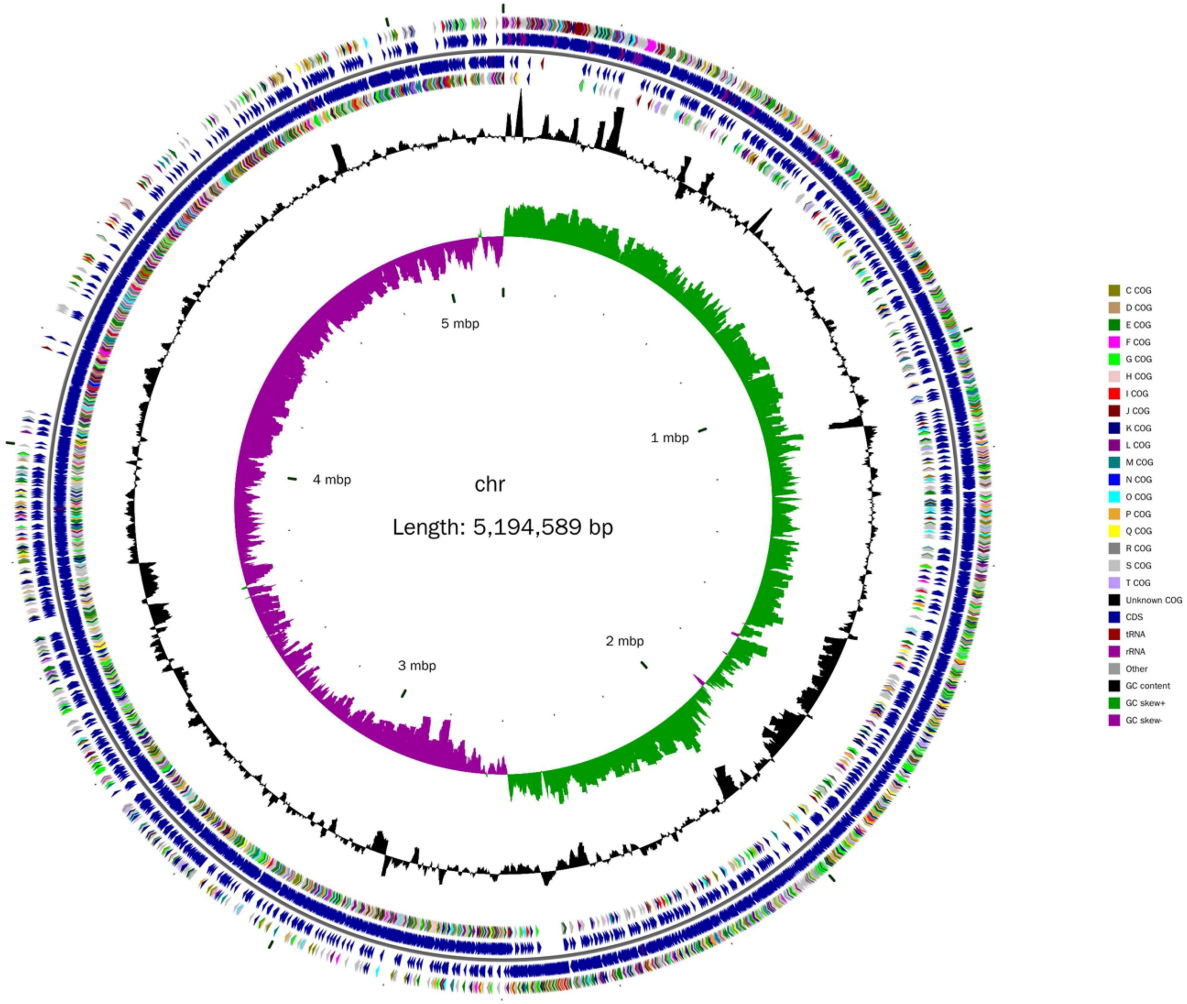
Circular chromosome diagram of *B. aryabhattai* LAD. From the inner circle to the outer circle, the information on each of the seven circles shows genome position, GC skew, GC content, coding sequence on the positive and strands (classified by Cluster of Orthologous Groups of protein), and the position of the coding sequence, tRNA and rRNA on the genome.

A total of 184,213 sequence reads was obtained in the next-generation sequencing. The *de novo* genome assembly using HGAP revealed a single circular chromosome genome and five circular plasmids in *B. aryabhattai* LAD. The newly sequenced genome was composed of 5,194,589 bp with a GC content of 38.12% (Figure 1). The five plasmids contained 212,484, 168,720, 137,532, 126,990, and 116,519 bp, respectively, with GC content ranging from 33.71 to 35.18%. The properties of the chromosome genome and plasmids are shown in Table 2. The genome contained 5,390 predicted genes, and the total length of the predicted genes was 4,303,422 genes accounting for 82.8% of the genome length. The five plasmids contained 246, 181, 165, 129, and 141 genes, respectively, with the coding percentage ranging from 66.7 to 73%. There were 112 tRNA genes and 39 rRNA genes on the chromosome genome. Fifteen genome islands (GIs), 4 CRISPRs, and 2 incomplete prophages were predicted on the genome of *B. aryabhattai* LAD. There were 13 5S rRNA genes (with an average length of 110 bp), 13 16S rRNA genes (average length of 1,549 bp), and 13 23S rRNA genes (average length of 2,932 bp) on the genome. The genomic annotation also revealed rRNA genes on plasmid1 and plasmid4. Specifically, one 5S rRNA gene of 108 bp was present on plasmid1, and there were one 5S rRNA gene (111 bp), one 16S rRNA gene (1,549 bp), and one 23S rRNA gene (2,932 bp) on plasmid4.

**Table 2:** Basic features of *B. aryabhattai* LAD chromosome genome and plasmids.

	Chromosome genome	Plasmid1	Plasmid2	Plasmid3	Plasmid4	Plasmid5
Length (bp)	5,194,589	212,484	168,720	137,532	126,990	116,519
GC content (%)	38.13	33.73	34.03	33.97	35.18	33.71
Gene number	5,390	246	181	165	129	141
Gene total length (bp)	4,303,422	141,816	123,078	96,090	88,983	79,167
Gene density (genes per kb)	1.038	1.158	1.073	1.2	1.016	1.21
Longest gene length (bp)	8,088	1,926	1,833	2,502	2,649	2,208
Gene average length (bp)	798.41	576.49	679.99	582.36	689.79	561.47
Gene length/genome (%)	82.84	66.74	72.95	69.87	70.07	67.94
GC content in gene region (%)	39.03	35.83	35.85	35.71	35.75	35.57
tRNA number	112	9	0	0	23	0
rRNA number	39	1	0	0	3	0

**Figure 2:**
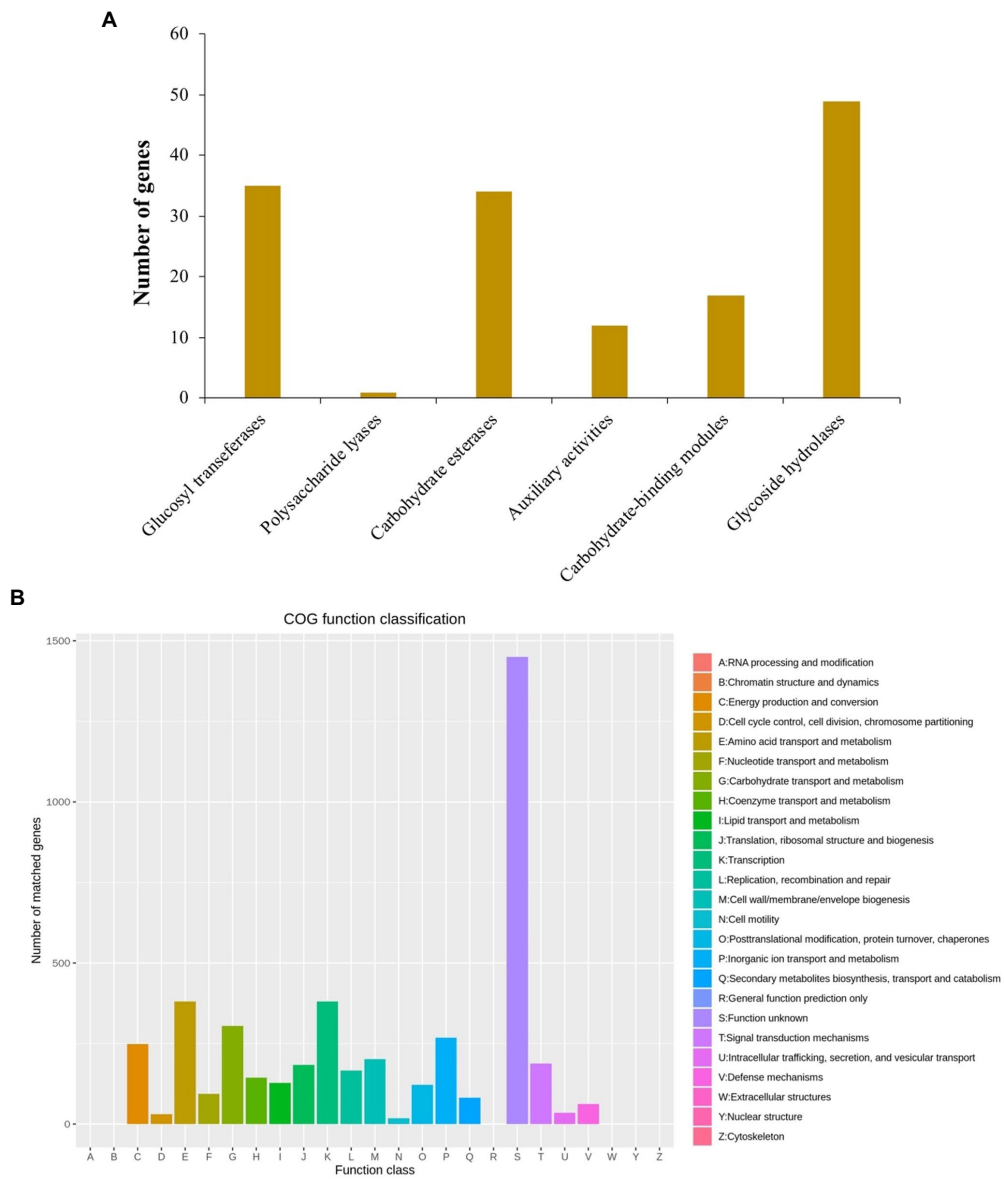
**(A)** Annotation of carbohydrate active enzymes (CAZymes) genes on *B. aryabhattai* LAD genome. The hmmscan program was used for estimation of the CAZy genes (alignment length > 80 amino acids, *E*-value <1e-5; alignment length < 80 amino acids, *E*-value <1e-3). **(B)** eggNOG annotation. The protein-coding gene sequences were compared to COG database using DIAMOND blastp (cutoff at 1e-6).

There were 148 CAZymes, including 35 glycosyltransferases (GTs), 1 polysaccharide lyases (PLs), 34 carbohydrate esterases (CEs), 12 auxiliary activities (AAs), 17 carbohydrate binding modules (CBMs), and 49 glycoside hydrolases (GHs) identified in the *B. aryabhattai* LAD chromosome genome *via* the CAZy annotation pipeline (Figure 2A). CAZymes were also present in two plasmids, with 1 GTs, 2 CEs, 2AAs, and 2GHs identified on plasmid 1, and 1 GTs and 1 GHs identified on plasmid 2. The eggNOG (evolutionary genealogy of genes: Non-supervised Orthologous Groups) annotation revealed an abundance of genes for basic cellular functions including amino acid transport and metabolism (381 genes, accounting for 7.07% of the total gene abundance), carbohydrate transport and metabolism (305, 5.7%), inorganic ion transport and metabolism (268, 5%), energy production and conversion (249, 4.6%), cell wall/membrane/envelope biogenesis (202, 3.7%), signal transduction mechanisms (188, 3.5%), and replication, recombination and repair (166, 3.1%; Figure 2B). The Gene Ontology (GO) classification revealed that ion binding, oxidoreductase activity, and DNA binding were very active molecular functions, and cellular nitrogen compound metabolic process, biosynthetic process, small molecule metabolic process, and transport were major biological processes (Figure 2B). The analysis against the Comprehensive Antibiotic Resistance Database (CARD) identified 28 antibiotic resistance genes and 4 antibiotic biosynthesis genes on the genome and 3 more antibiotic resistance genes on the plasmids.

### Dig of promoting growth genes

In the whole genome of LAD strain, there were many genes related to the promotion of plant growth, some of which are shown in the Table 3. Ten related genes were found to be involved in the Nitrogen metabolism pathway in LAD strains, and the *NifF* gene was found at chr_4496 and *NifU* gene at chr_5091. In the KEGG pathway of Tryptophan metabolism, 25 genes related to IAA synthesis were identified, including amidase *(amiE)*, which hydrolyzes indole-3-acetamide to indoleacetic acid, and aldehyde dehydrogenase *(ALDH)*, which hydrolyzes indole-3-acetaldehyde to indoleacetic acid. We identified 20 genes related to biofilm formation. Four genes encoding related proteins in LAD strains mapped to the KEGG pathway of phosphonate and phosphinate metabolism, including phosphoenolpyruvate phosphomutase *(pepM)*, phosphonopyruvate decarboxylase, phosphoribosyl 1,2-cyclic phosphate phosphodiesterase (*phnP*) and phosphinothricin acetyltransferase (*pat*, *bar*), and a total of 53 phosphatase genes were identified in the LAD genome, mapping to 34 KEGG metabolic pathways. The extracellular polysaccharide production by LAD strains is closely related to carbohydrate metabolism, a total of 315 carbohydrate related genes were found for carbohydrate metabolism. The two-component regulatory system is a major mechanism for biotransduction and response to external environmental changes, which can sense environmental changes, regulate internal gene expression and play an important role in the survival and adaptation of microorganisms to different environments, and help microorganisms preserve their competitive advantage in the environment. A total of 98 KEGG pathways of two-component system were identified in LAD strains, including phosphate limitation, oxygen limitation, and temperature limitation system. A total of 106 genes related to the ABC transporters metabolic pathway were identified in LAD strains, including mineral and organic ion transporters, phosphate and amino acid transporters, peptide and nickel transporters, monoasccharide transporters, oligosaccharide, polyol, lipid transporters, metallic cation and iron-siderop hore and vitamin B12 transporters.

**Table 3:** Genes related to the promotion of plant growth(partial).

KEGG pathway_ ID	Gene_ ID	Annotation information
K03839	chr_4996	*fldA*,*nifF*,*isiB*;flavodoxin І
K04488	chr_5091	*iscU*,*nifU*;nitrogen fixation protein *NifU* and related proteins
K01426	chr_999	E3.5.1.4, *amiE*; amidase
K00128	chr_2221	*ALDH*; aldehyde dehydrogenase (NAD+)
K01841	chr_754	*pepM*; phosphoenolpyruvate phosphomutase
K09459	chr_755	E4.1.1.82; phosphonopyruvate decarboxylase
K06167	chr_2553	*phnP*; phosphoribosyl 1,2-cyclic phosphate phosphodiesterase
K03823	chr_2685	*pat*; phosphinothricin acetyltransferase
K07658	chr_3082	*phoB1*, *phoP*; two-component system, *OmpR* family, alkaline phosphatase synthesis response regulator *PhoP*
K07658	chr_4881	*phoB1*, *phoP*; two-component system, *OmpR* family, alkaline phosphatase synthesis response regulator *PhoP*
K01113	chr_5,198	*phoD*; alkaline phosphatase D

### Comparative genomics analyses

The ANI between the whole genome sequences was measured by pairwise genome comparisons using JSpeciesWS Online Service to evaluate the homologies of the whole genomes and determine if the strains belong to the same species. The matrix of nucleotide identities between the whole genomes of the *Bacillus* strains was shown in Table 4. The ANI value of *B. aryabhattai* LAD based on BLAST against other *Bacillus* strains ranged from 67.7 to 95.6%, and the aligned percentage of the genome sequence ranged from 24.2 to 82.4%. The ANI values of *B. aryabhattai* LAD compared with other *B. aryabhattai* strains and *B. megaterium* strains were higher than 95%, which was the threshold identity for species boundaries.

**Table 4:** Average nucleotide identities (ANI) analysis for pairwise genome comparison between *B. aryabhattai* LAD and other *Bacillus* strains.

	B8W22	DSM 319	NCT-2	AB 211	B14	AFS07 5,785	N11	ATCC 14581	AM1 (2019)	FJ-6	BIM B-1314D	S00 060	G25-109	N11	LAD
*B. aryabhattai* B8W22	*	99.94	99.81	99.96	99.92	99.94	99.92	99.84	79.19	99.95	99.8	99.79	99.93	85.11	99.77
*B. megaterium* DSM319	99.94	*	99.88	99.93	99.87	99.92	99.95	99.9	79.09	99.93	99.86	99.81	99.94	85.39	99.83
*B. megaterium* NCT-2	99.81	99.88	*	99.86	99.64	99.77	99.85	99.96	79.6	99.78	99.95	99.91	99.9	86.24	99.91
*B. aryabhattai* AB211	99.96	99.93	99.86	*	99.89	99.93	99.91	99.87	79.34	99.93	99.84	99.83	99.93	85.21	99.82
*B. aryabhattai* B14	99.92	99.87	99.64	99.89	*	99.93	99.89	99.68	79.18	99.93	99.59	99.55	99.82	84.43	99.56
*B. aryabhattai* AFS075785	99.94	99.92	99.77	99.93	99.93	*	99.93	99.78	79.35	99.98	99.73	99.71	99.89	84.85	99.69
*B. cereus* group sp. N11	99.92	99.95	99.85	99.91	99.89	99.93	*	99.87	79.07	99.94	99.82	99.768	99.92	85.39	99.78
*B. megaterium* ATCC 14581	99.84	99.9	99.96	99.87	99.68	99.78	99.87	*	79.27	99.81	99.95	99.901	99.91	86.43	99.91
*Bacillus sp.* AM1(2019)	79.19	79.09	79.6	79.34	79.18	79.35	79.07	79.27	*	79.17	79.19	79.159	78.95	67.27	78.89
*B. aryabhattai* FJ-6	99.95	99.93	99.78	99.93	99.93	99.98	99.94	99.81	79.17	*	99.74	99.717	99.89	85	99.71
*B. megaterium* BIM B-1314D	99.8	99.86	99.95	99.84	99.59	99.73	99.82	99.95	79.19	99.74	*	99.96	99.91	86.29	99.95
*B. aryabhattai* S00060	99.79	99.81	99.91	99.83	99.55	99.71	99.77	99.9	79.16	99.72	99.96	*	99.91	86.17	99.93
*B. aryabhattai* G25-109	99.93	99.94	99.9	99.93	99.82	99.89	99.92	99.91	78.945	99.89	99.91	99.91	*	85.47	99.87
*B. cereus* group sp. N11	85.11	85.39	86.24	85.21	84.43	84.85	85.39	86.43	67.27	85	86.29	86.17	85.47	*	86.49
*B. aryabhattai* LAD	99.77	99.83	99.91	99.82	99.56	99.69	99.78	99.91	78.89	99.71	99.95	99.93	99.87	86.49	*

Asterisk indicates the same strain.

**Figure 3:**
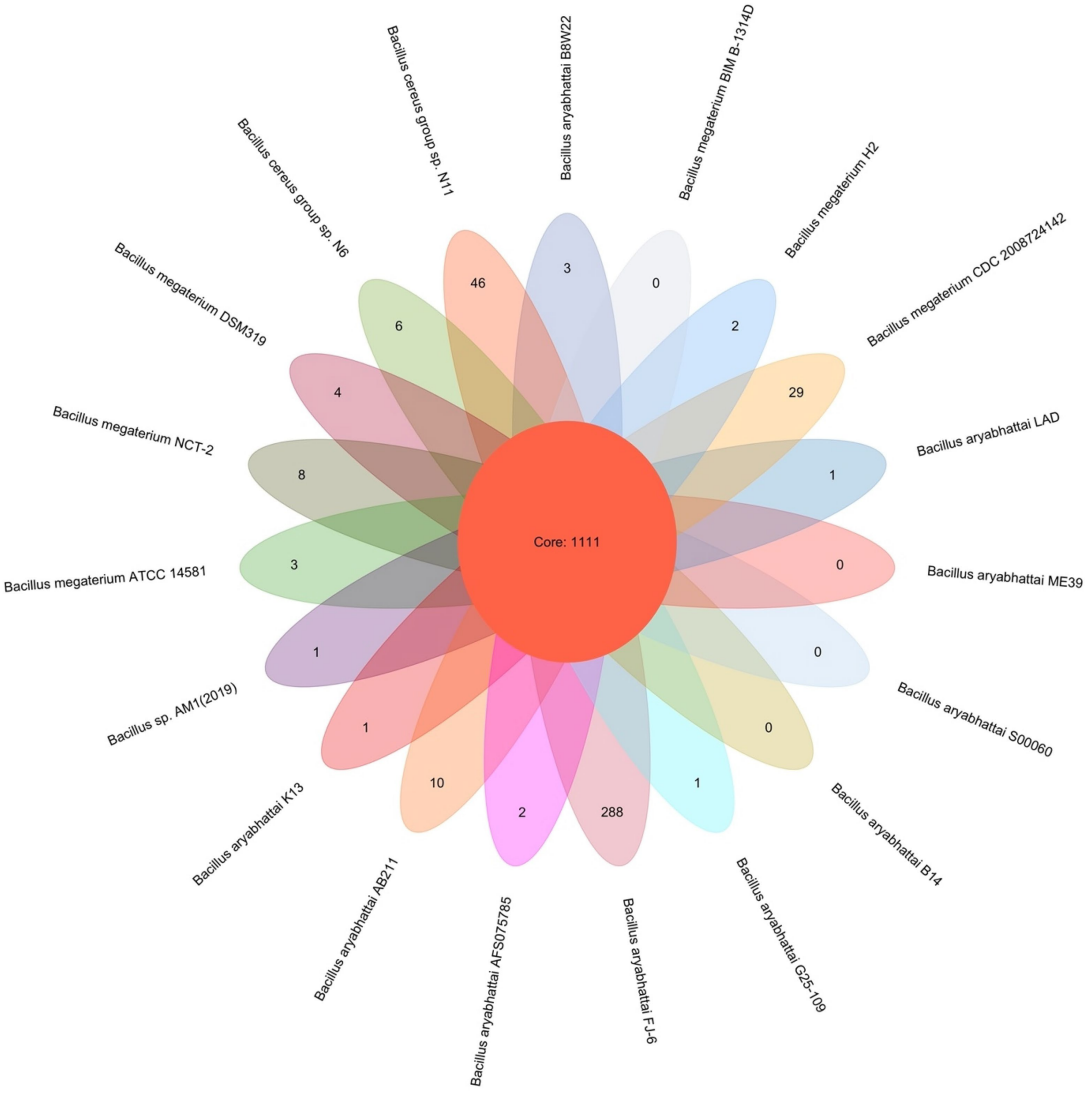
Pan-genome analysis of 19 *Bacillus* strains. The number in the overlapping center represents the number of orthologous coding sequences (core genome) shared by all analyzed strains. The number of coding sequences specific to each strain was shown in the non-overlapping portion of the oval.

PGAP pipeline was used to determine the pan-genome for *B. aryabhattai* LAD and 18 other *Bacillus* strains. The 19 compared *Bacillus* strains had a total pan-genome consisted of 94,898 putative protein-coding genes, and 1,111 of them (accounting for 1.17% the pan-genome) were core conserved genes across the genomes of the 19 strains (Figure 3). The number of strain-specific genes for each train ranged from 0 to 288, and *Priestia aryabhattai* FJ-16 had 288 strain-specific genes which was the highest among the 20 strains. The isolate in this study, *B. aryabhattai* LAD, had 1 strain-specific gene.

**Figure 4:**
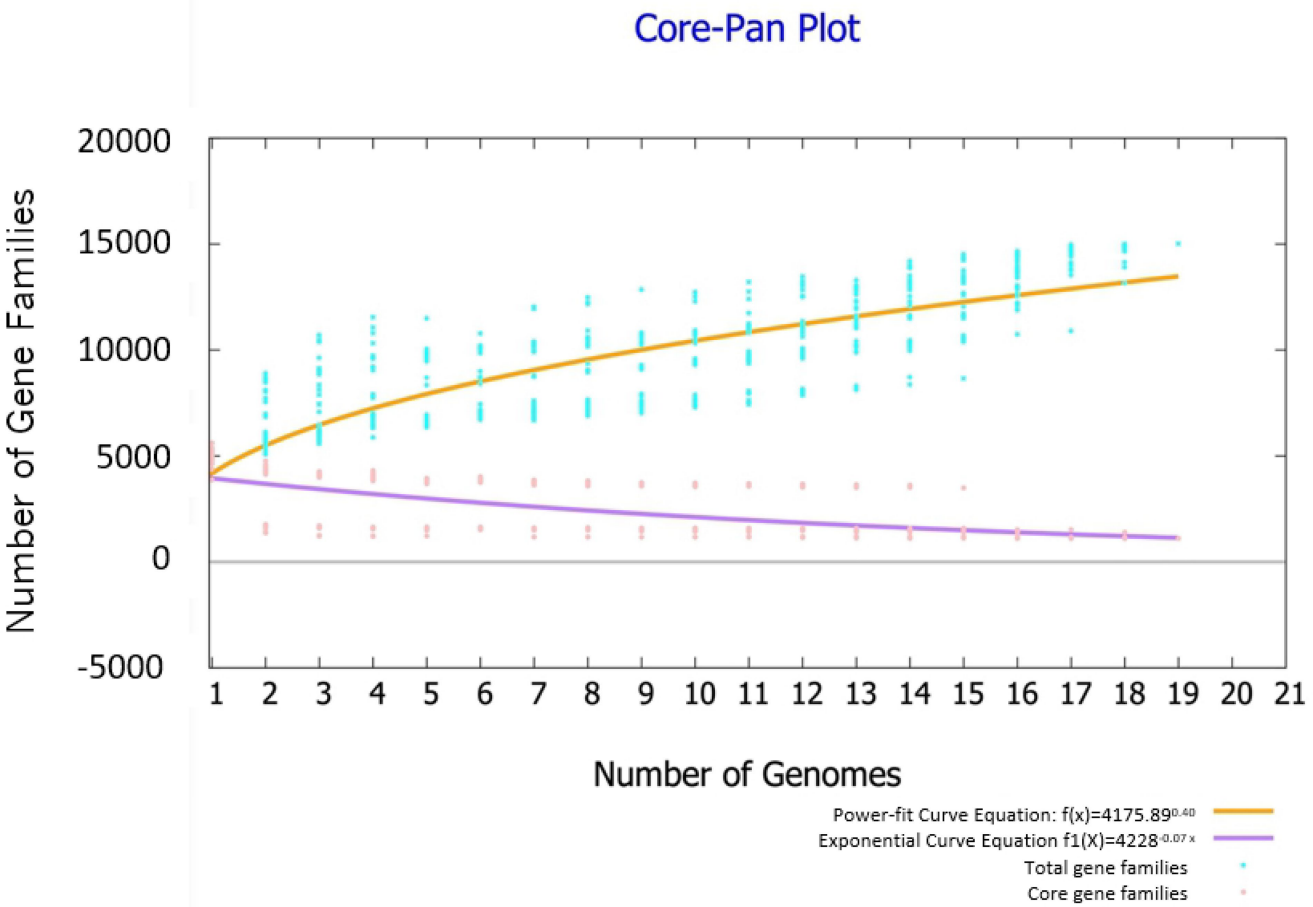
Pan genome and core genome profile plot of 19 *Bacillus* strains. The analysis was performed through the Bacterial Pan Genome Analysis (BPGA) software package.

Highly reliable pan-genome analysis can be obtained by mathematical extrapolation with more than five genomes (Vernikos et al., 2015). The analysis *via* BPGA showed that the pan-genome of the 19 *Bacillus* genomes had a parameter γ of 0.4 in the reduced power-fit curve equation [f(x) = 4175n0.4] suggesting that the pan-genome was open (Figure 4). The exponential curve equation of core genome [f1(x) = 4228.01e-0.07] had a steep slope and reached a minimum of 1,111 gene families.

### *Bacillusaryabhattai* LAD growth and production of IAA and EPSs

**Figure 5:**
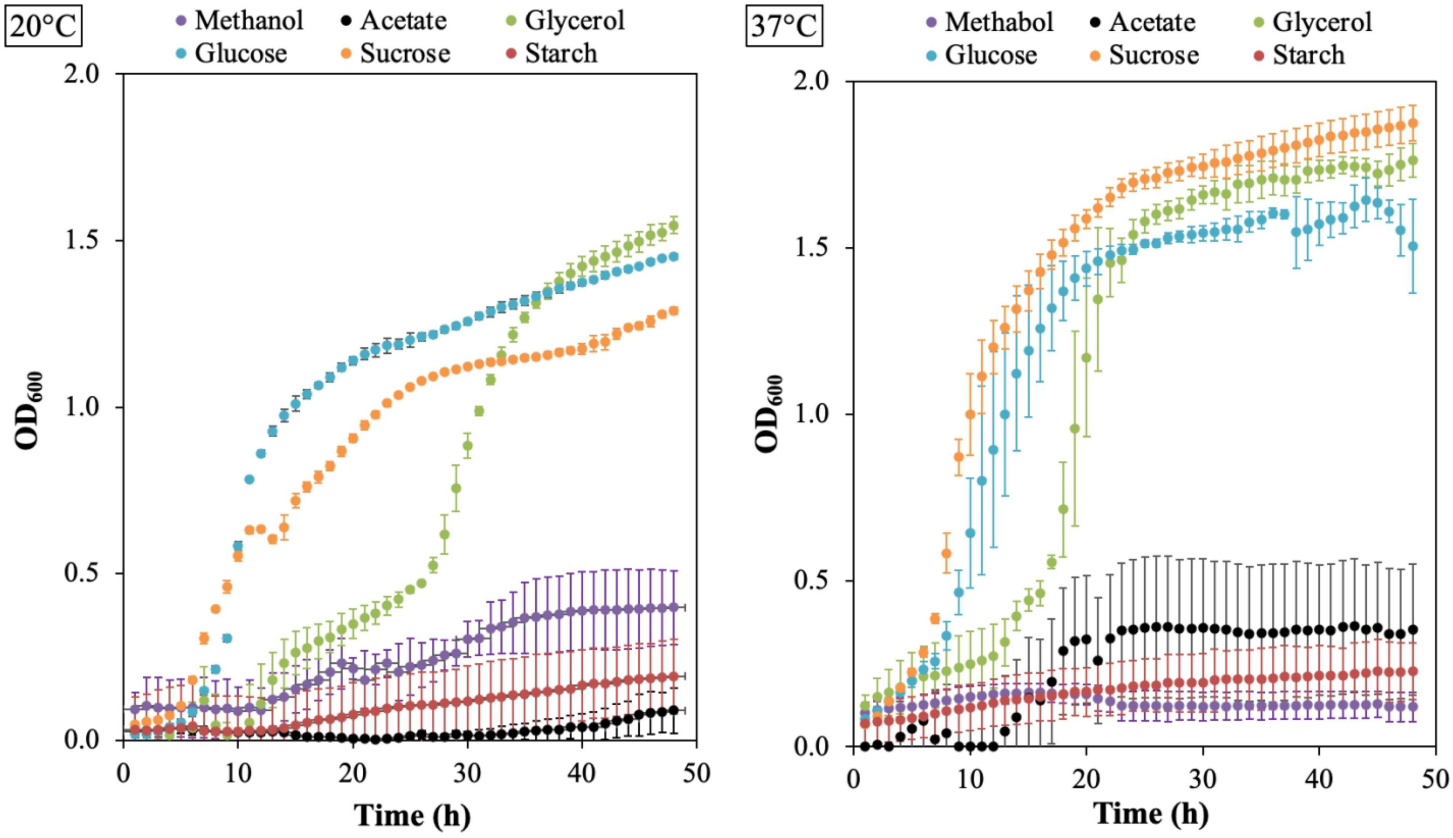
The growth plots for *B. aryabhattai* LAD. LAD cells were cultivated in the optimum growth medium with different carbon sources, i.e., methanol (0.5, v v^−1^), sodium acetate (2%), glycerol (2%), glucose (2%), sucrose (2%), and starch (2%), respectively. The temperature for LAD growth included 20°C and 37°C. Each data point represents the mean value of three replicates.

The growth of *B. aryabhattai* LAD on different carbon sources was evaluated with Epoch 2 microplate spectrophotometer at 20°C and 37°C. The results showed that LAD can utilize all the six carbon sources, including acetate, glycerol, glucose, sucrose, starch, and methanol, under appropriate temperatures (Figure 5). LAD had the best growth on sucrose at 37°C, and the bacterial density reached the highest OD_600_ value (as high as 1.9). Glucose and glycerol supported good LAD growth both at 20°C and 37°C, with the OD_600_ value of glucose reaching 1.4 at 20°C and 1.5 at 37°C, and the OD_600_ value of glycerol reaching 1.1 at 20°C and 1.8 at 37°C. Interestingly, LAD can efficiently utilize methanol as carbon source for growth with the ability to reach a high bacterial density (an OD_600_ value of 0.4 at 20°C and 0.2 at 37°C) which demonstrates that *B. aryabhattai* LAD belongs to methylotrophic bacteria. Acetate also supported the growth of LAD with the bacterial cell density reaching an OD_600_ of 0.4 at 37°C. LAD was able to utilize starch, but the bacterial growth rate and final cell density were both lower than using other carbon sources.

**Figure 6:**
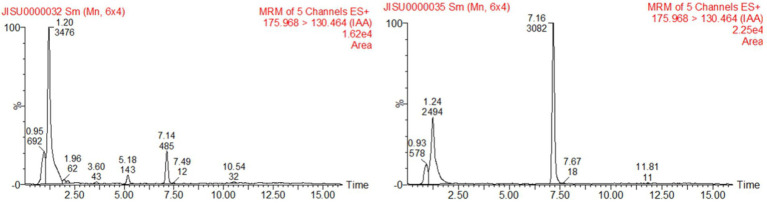
Detection of IAA production by UPLC. The left figure shows the sample of the control, and the right shows the sample of *B. aryabhattai* LAD culture cultivated with maize seedlings.

To examine the IAA-producing capacity of LAD, maize seedlings were cultivated with LAD cell suspensions (10^5^ CFU ml^−1^) and with the same volume of sterile water, respectively, for 8 days. The concentrations of IAA in the bacterial suspensions and water were measured with UPLC. The concentration of IAA in the LAD suspension was 0.191 ± 0.014 μg ml^−1^, which was significantly higher than that in the hydroponic system without LAD (0.036 ± 0.0096 μg ml^−1^; *t*-test, *p* < 0.001; Figure 6). The results showed that LAD can produce IAA or promote IAA secretion from maize roots. The test of EPSs in the LAD cultures showed that stable production of EPSs by LAD was only detected in medium with sucrose as the sole carbon source. The EPSs production in sucrose supplemented medium was 3.18 ± 0.0198 g L^−1^ at 24 h and 0.63 ± 0.0041 g L^−1^ at 72 h.

### Impacts on maize root development

**Figure 7:**
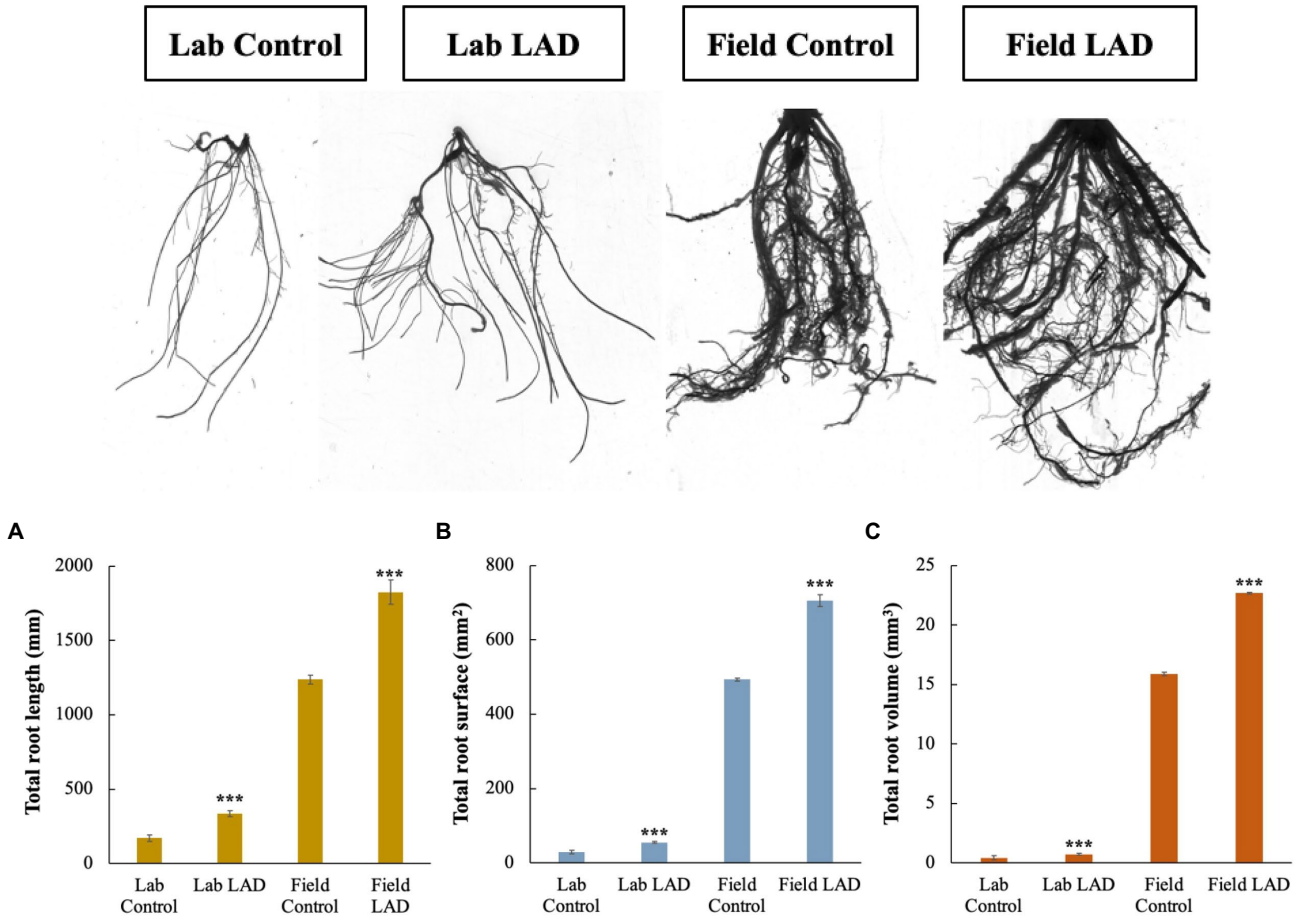
Maize root development in laboratory and field soils impacted by *B. aryabhattai* LAD. **(A)** The impacts of LAD on the maize root length. **(B)** The impacts of LAD on the maize root surface area. **(C)** The impacts of LAD on the maize root volume. Lab control: the root of the maize seedlings treated with water and cultivated in laboratory soil for 14 d; Lab LAD: the root of the maize seedlings treated with LAD and cultivated in laboratory soil for 14 d; Field control: the root of the maize seedlings treated with water and cultivated in laboratory soil for 60 d; Field LAD: the root of the maize seedlings treated with LAD and cultivated in laboratory soil for 60 d. Each treatment had 10 maize plants and was repeated three times. Student’s *t*-test was used to compare the root development under LAD treatment with that of the control. Statistical differences were shown with asterisks (i.e., *** representing *p* < 0.001).

To investigate how LAD affects the maize root development, the maize seeds were treated with 10^5^ CFU ml^−1^ LAD cell suspensions and grown in laboratory and filed soils, respectively. The roots were collected and analyzed with root analyzer. The laboratory cultivation results showed that the total root length, total root surface area and total root volume of the LAD treatment group is 97.0, 90.1, and 75.6% higher than that of the control, respectively (*t*-test, *p* < 0.001; Figure 7). Like the laboratory cultivation, the results of field experiments revealed an increase of 47.6, 43.1, and 42.9% in the total root length, surface area, and volume in the LAD treatment compared with the control (*t*-test, *p* < 0.001). The longest root in the LAD treatment was 197% longer than the longest root in the control. The experiments demonstrated that LAD treatment significantly increased the maize root length, surface area, and volume (*p* < 0.001).

## Discussion

Plant-microbiome interactions are critical for plant health and growth. Rhizosphere microbes reside near or on the plant root and have direct contact and intense interactions with the plant tissue, therefore the root microbiome can utilize root exudates and secrete compounds that remarkably influence plant growth (Haney et al., 2015; Pascale et al., 2020). The interactions between plant and rhizosphere microbes are extremely complex, and the mechanism, ecological significance, and potential for application remain to be clarified. The data in this study shed light on the mechanism of *B. aryabhattai* LAD promoting plant growth based on both genomic analysis and experimental evidence. Whole genome sequencing of *B. aryabhattai* LAD showed that the rhizobacteria had diverse pathways for carbohydrate metabolism and mechanisms for environmental adaptations. The growth experiments of *B. aryabhattai* LAD demonstrated that LAD cells can utilize different types of carbons sources ranging from one-carbon methanol to polymeric carbohydrate starch, and the LAD cells can also grow well in a wide range of temperatures (20°C to 37°C). These properties are important for LAD to adapt to the fluctuating conditions in soil environments and further forming possible symbiosis with plant hosts, as plant root secretion of different carbon chain length may serve as nutrient supply for the root-associated bacteria while the bacterial activities may facilitate plant performance (Blom et al., 2011; Cheng et al., 2019; Lucke et al., 2020).

IAA, one of the most common and physiologically active plant hormones in nature, can induce cell elongation and division and is important for plant development and growth. Bacterial production of IAA as plant growth promoting strategy has been demonstrated in previous studies (Mohite, 2013; Wagi and Ahmed, 2019). For example, the IAA production by *B. cereus* So3II and *B. subtilis* Mt3b was shown by, and the growth conditions of the two *Bacillus* strains could be optimized to enhance optimum bacterial growth and production of IAA which ultimately may be used for plant growth stimulation. *B. aryabhattai* LAD in the present study produced high yield of IAA when cultivated with maize plant suggesting the symbiosis of the rhizobacteria with maize plant. Besides IAA production, *B. aryabhattai* LAD can also produce EPSs which is another plant growth-promoting trait (Wagi and Ahmed, 2019). A recent study by showed that EPS-producing bacteria stimulated the salt tolerance of plant because the bacterial produced EPSs can bind cations significantly decreasing the Na^+^ content in the environment (Upadhyay et al., 2011). Interestingly, the EPSs production decreased from 3.18 g L^−1^ at 24 h to 0.63 g L^−1^ at 72 h, suggesting that the produced EPSs might be utilized by LAD cells when available nutrients are depleted.

Microbial metabolisms associated with one-carbon compound conversion were prevalent in rhizosphere, and the methanol metabolism by *B. aryabhattai* LAD may contribute to the rhizosphere nutrient cycling (Knief et al., 2012; Li et al., 2019). The genome annotation revealed that *B. aryabhattai* LAD genome contains genes of alcohol dehydrogenases, and the alcohol dehydrogenases enzymes can facilitate the conversion of alcohols to aldehydes with production of NADH. Aldehyde dehydrogenase genes were also detected on *B. aryabhattai* LAD genome, and the products of the genes catalyze the oxidation of the aldehydes generated from methanol. The other possible pathway for methanol metabolism in *B. aryabhattai* LAD was through alcohol oxidase (AOX), catalyzing the oxidation of methanol to formaldehyde with production of hydrogen peroxide, and catalase, breaking down hydrogen peroxide, as genes for AOX and peroxidase were detected on *B. aryabhattai* LAD genome. Plant roots secrete a variety of carbon-containing compounds which significantly influence rhizobacterial activities and community structure (O’Neal et al., 2020; Okutani et al., 2020; Liang et al., 2021). The root exudates of varied molecular weight and complexity may serve as organic carbon sources for microbial growth, and the ability for rhizobacteria utilizing root exudates is critical for both the bacterial populations and the host plant (Mark et al., 2005; Fethel et al., 2014; Olanrewaju et al., 2019). The CAZymes annotation of *B. aryabhattai* LAD genome revealed an abundance of genes associated with the assimilation, modification, and decomposition of carbohydrates.

The whole genome of *B. aryabhattai* LAD was obtained *via* next-generation sequencing, and the comparative analysis showed that *B. aryabhattai* LAD had high similarities to other *B. aryabhattai* strains and strains in *B. megaterium*. For genomic characterization of *B. aryabhattai* LAD, the pan and core genome analysis were performed to cluster the genes in all the examined genomes. The eggNOG annotation and GO annotation revealed large groups of genes associated with carbohydrate transport and metabolism, oxidoreductase activity, cellular nitrogen compound metabolic process, and biosynthetic process, all of which are important for the symbiotic relationship between bacterial populations and plants and were also found in other plant growth promoting bacterial strains (Taghavi et al., 2010; Han et al., 2011; Bhattacharyya et al., 2017). The CARD analysis identified 31 antibiotic resistance genes and 4 antibiotic biosynthesis genes on the genome and plasmids of *B. aryabhattai* LAD, suggesting that *B. aryabhattai* LAD can be resistant to various antibiotics and have potential for synthesizing antibiotics. Antibiotics production were also reported in other *Bacillus* strains which has a major role for rhizobacteria to suppress plant diseases. For example, the whole genome sequencing of *B. amyloliquefaciens* FZB42 showed a high potential for production of polyketides bacillaene and difficidin, and over 8.5% of the genome is committed antibiotics and siderophores biosynthesis (Chen et al., 2007). Kinsella et al. (2008) also showed a high yield of antibiotics (i.e., surfactin and iturin) by *Bacillus subtilis* on cucumber roots (Kinsella et al., 2008).

## Conclusion

Plant roots harbor a diverse microbial community, and the complicated interactions between plant and rhizosphere microbes have major ecological importance and critical implications for agricultural practices. The whole genome sequencing of methylotrophic *B. aryabhattai* LAD revealed many signature genes that are functionally associated with plant growth promotion. Genome analyses revealed the ability of *B. aryabhattai* LAD to adapt to the environments with antibiotics, oxidative, cold temperature, and heavy metal stresses. Our experiments confirmed that *B. aryabhattai* LAD has versatile metabolism and can utilize a wide range of carbon sources. *B. aryabhattai* LAD is also capable of synthesizing IAA and EPSs that are beneficial for plant growth and bacterial colonization. The genomics analysis and experimental studies collectively demonstrated the plant growth promoting capacities of *B. aryabhattai* LAD making it an exceptional bacterial strain for application in agricultural practices. The impacts of *B. aryabhattai* LAD on maize root development was directly evaluated, and the results demonstrated the root development stimulation and plant growth promoting capacities of the *Bacillus* strain. Future efforts are needed for studying the molecular mechanisms of *B. aryabhattai* LAD in promoting plant growth and evaluating the ecological impacts of bioaugmentation with LAD on soil microbial community.

## Data availability statement

The datasets presented in this study can be found in online repositories. The names of the repository/repositories and accession number(s) can be found in the article/supplementary material.

## Ethics statement

Study protocols of plant materials comply with the IUCN Policy Statement on Research Involving Species at Risk of Extinction and the Convention on the Trade in Endangered Species of Wild Fauna and Flora.

## Author contributions

NZh and BL conceived and designed the study. CD conducted the experiments. CD, XW, NZe, and XL performed data analysis and prepared the figures. XL wrote the main manuscript. All authors contributed to the article and approved the submitted version.

## Funding

This work was supported by the National Key Research and Development Program of China (2017YFD0200807), the Liaoning Province Rural Science and Technology Special Action Project (2022-09), and Shenyang Science and Technology Project (20-207-3-35).

## Conflict of interest

The authors declare that the research was conducted in the absence of any commercial or financial relationships that could be construed as a potential conflict of interest.

## Publisher’s note

All claims expressed in this article are solely those of the authors and do not necessarily represent those of their affiliated organizations, or those of the publisher, the editors and the reviewers. Any product that may be evaluated in this article, or claim that may be made by its manufacturer, is not guaranteed or endorsed by the publisher.
